# Identification of Sclerostin as a Putative New Myokine Involved in the Muscle-to-Bone Crosstalk

**DOI:** 10.3390/biomedicines9010071

**Published:** 2021-01-12

**Authors:** Maria Sara Magarò, Jessika Bertacchini, Francesca Florio, Manuela Zavatti, Francesco Potì, Francesco Cavani, Emanuela Amore, Ilaria De Santis, Alessandro Bevilacqua, Luca Reggiani Bonetti, Pietro Torricelli, Delphine B. Maurel, Stefano Biressi, Carla Palumbo

**Affiliations:** 1 Department of Biomedical, Metabolic and Neural Sciences, Section of Human Morphology, University of Modena and Reggio Emilia, 41124 Modena, Italy; mariasara.magaro@unimore.it (M.S.M.); manuela.zavatti@unimore.it (M.Z.); francesco.cavani@unimore.it (F.C.); Emanuela.amore@unimore.it (E.A.); carla.palumbo@unimore.it (C.P.); 2 Department of Cellular, Computational and Integrative Biology (CIBIO) and Dulbecco Telethon Institute, University of Trento, 38123 Povo, Trento, Italy; francesca.florio@unitn.it (F.F.); stefano.biressi@unitn.it (S.B.); 3 Department of Medicine and Surgery—Unit of Neurosciences, University of Parma, 43126 Parma, Italy; francesco.poti@unipr.it; 4 Department of Medical and Surgical Sciences (DIMEC), Alma Mater Studiorum, University of Bologna, 40138 Bologna, Italy; i.desantis@unibo.it; 5 Advanced Research Center for Electronic Systems (ARCES), University of Bologna, 40126 Bologna, Italy; alessandro.bevilacqua@unibo.it; 6 Department of Medical and Surgical Sciences for Children and Adults, AOU Policlinico of Modena, University of Modena and Reggio Emilia, 41124 Modena, Italy; luca.reggianibonetti@unimore.it; 7 Department of Radiology, University Hospital of Modena, 41124 Modena, Italy; pietro.torricelli@unimore.it; 8 Pharmaceutical Sciences Department, University of Bordeaux, BioTis, INSERM Unit 1026, 33076 Bordeaux, France; delphine.maurel@u-bordeaux.fr

**Keywords:** sclerostin, muscle-to-bone crosstalk, myokine

## Abstract

Bone and muscle have been recognized as endocrine organs since they produce and secrete “hormone-like factors” that can mutually influence each other and other tissues, giving rise to a “bone–muscle crosstalk”. In our study, we made use of myogenic (C2C12 cells) and osteogenic (2T3 cells) cell lines to investigate the effects of muscle cell-produced factors on the maturation process of osteoblasts. We found that the myogenic medium has inhibitory effects on bone cell differentiation and we identified sclerostin as one of the myokines produced by muscle cells. Sclerostin is a secreted glycoprotein reportedly expressed by bone/cartilage cells and is considered a negative regulator of bone growth due to its role as an antagonist of the Wnt/β-catenin pathway. Given the inhibitory role of sclerostin in bone, we analyzed its expression by muscle cells and how it affects bone formation and homeostasis. Firstly, we characterized and quantified sclerostin synthesis by a myoblast cell line (C2C12) and by murine primary muscle cells by Western blotting, real-time PCR, immunofluorescence, and ELISA assay. Next, we investigated in vivo production of sclerostin in distinct muscle groups with different metabolic and mechanical loading characteristics. This analysis was done in mice of different ages (6 weeks, 5 and 18 months after birth) and revealed that sclerostin expression is dynamically modulated in a muscle-specific way during the lifespan. Finally, we transiently expressed sclerostin in the hind limb muscles of young mice (2 weeks of age) via in vivo electro-transfer of a plasmid containing the *SOST* gene in order to investigate the effects of muscle-specific overproduction of the protein. Our data disclosed an inhibitory role of the muscular sclerostin on the bones adjacent to the electroporated muscles. This observation suggests that sclerostin released by skeletal muscle might synergistically interact with osseous sclerostin and potentiate negative regulation of osteogenesis possibly by acting in a paracrine/local fashion. Our data point out a role for muscle as a new source of sclerostin.

## 1. Introduction

The musculoskeletal system consists principally of bones, muscles, tendons, ligaments, and articular cartilage. These are arranged throughout the body and physically and mechanically interact to assure the integrated motor activity and the musculoskeletal metabolism/homeostasis [1]. In particular, the musculoskeletal system’s primary function includes supporting the body, allowing movement, and protecting vital organs. In this system, bones relate to other bones and muscles via connective tissue such as tendons and ligaments. Bones provide stability and muscles keep bones in place and allow the motor activity. Moreover, the skeletal portion of the system has the role to store calcium and phosphorus and contains the hematopoietic system [2].

There are several diseases and disorders that may affect this system, and often they are very difficult to diagnose due to the close relation of the musculoskeletal system to other internal systems. Musculoskeletal conditions range from those that arise in an acute manner and are short-lived, such as fractures, to lifelong pathologies associated with chronic pain and disability such as genetic diseases (achondroplasia, osteogenesis imperfecta, muscular dystrophy, and myopathies) [3,4], metabolic diseases such as osteoporosis and osteopenia [5], tumors of the bones and the muscle such as osteosarcoma and rhabdomyosarcoma [6], joint diseases such as osteoarthritis and rheumatoid arthritis [7], and aging in general characterized by bone and muscle weakness (osteopenia and sarcopenia) [8,9]. All these diseases comprise dysregulation of the physiology of all the tissues among the musculoskeletal system; therefore, a better understanding of the molecular mechanisms responsible for the crosstalk among these tissues is needed. In particular, the condition related to musculoskeletal aging is one of the major public health interests and is typical of the demographic changes in the population. It is associated with a high risk of fractures and joint and muscle hypofunctionality with consequent loss of autonomy in elderly people, and this condition is therefore correlated with high morbidity and healthcare rates [10,11]. Sarcopenia and osteoporosis are linked and commonly associated with aging, often leading to a frailty syndrome, a pathological condition characterized by a general decline, which includes multifactorial causes [12,13].

During the last decade, bone and muscle were increasingly recognized as interacting tissues, not only because of their proximity and their integrated function for motor activity, but also for the fact to be recognized as endocrine target tissues and to be endocrine organs themselves [14]. In fact, the two tissues interact with each other by paracrine and endocrine signals and modulate their mutual development and function since intrauterine life to oldness [15]. The muscle–bone crosstalk is supported by preclinical and clinical data, showing the presence of many tissue-specific factors released by osteoblasts and osteocytes, including prostaglandin E_2_, osteocalcin, and IGF-1, which have a potential impact on skeletal muscle cells. Moreover, a growing number of muscle-released factors with bone-modulating properties have been identified. These include insulin-like growth factor-1 (IGF-1), fibroblast growth factor-2, IL-6, IL-15, myostatin, osteoglycin, irisin, and osteoactivin, which have been initially implicated in the pathogenesis of sarcopenia but appear to also be regulators of bone remodeling and thus potentially relevant for osteoporosis [16,17,18,19,20,21,22]. However, despite this recent effort, limited data exist about the molecular basis controlling the muscle-to-bone crosstalk.

In our study, for the first time, we clearly identified a new putative myokine, sclerostin, that until recently had been described as a potent inhibitor of bone formation produced mainly by osteocytes [23,24]. Our data demonstrate the expression of sclerostin both in muscle cells in vitro and in muscles collected from variously aged mice and differing in metabolic and load-bearing features. Moreover, through a functional approach, we investigated the role of muscle-originated sclerostin in bone formation. In conclusion, this study highlights, for the first time, skeletal muscle as a new source of sclerostin. Sclerostin produced at the muscular level may act in combination with bone sclerostin in severe conditions of increased bone fragility. This discovery is particularly relevant for the development of treatments for pathophysiological conditions characterized by simultaneous bone and muscle loss, such as aging-associated osteoporosis/sarcopenia or forced immobilization. It is of note that this new finding could also lead to new paths in the knowledge of the mechanisms underlying genetic diseases such as neuromuscular diseases or cancers where both muscle and bone tissues are severely affected. Targeting pathways that regulate bone and muscle systemically and/or through the manipulation of more localized pathways that facilitate communication between the two tissues are the privileged directions for the identification of new molecules, which could simultaneously prevent, reduce, or restore bone and muscle age-related deterioration.

## 2. Materials and Methods

### 2.1. Cell Cultures

C2C12 myoblasts (CRL-1772; ATCC) were cultured in a growth medium (GM) composed of DMEM containing 10% FBS, 1% penicillin/streptomycin (P/S), and 2 mM L-glutamine in a 5% CO_2_ atmosphere at 37 °C. To induce myogenic differentiation, the cells were plated on tissue culture plates and grown to 95% confluence before switching to the differentiation medium (DM) (DMEM with 5 μg/mL insulin). The cells were replenished with fresh DM every 48 h to induce differentiation until 14 days.

Osteoblastic cell line 2T3 provided by Lynda Bonewald (Indiana University School of Medicine, Indianapolis, IN, USA) was cultured in a complete medium composed of a MEM supplemented with 10% FBS, 2 mM L-glutamine, and 1% P/S. For osteogenic differentiation, a complete medium supplemented with 50 μg/mL ascorbic acid and 4 mM β-glycerophosphate was used. The differentiation medium was changed every two days of culture.

C57 primary myoblasts were isolated from limb muscles of young (14–30 days old) mice previously injured with a 30-gauge needle and dissociated to isolate pure populations of myoblasts as described previously [25]. Primary cultures were plated on 5 μg/mL laminin-1/collagen-coated dishes and amplified in a growth medium (GM) consisting of Ham’s F-12 with 20% FBS, 2.5 ng/mL basic fibroblast growth factor, 2 mM L-glutamine, and 1% P/S.

To induce differentiation, myoblast cultures were seeded on coated dishes at 60% confluence and the growth medium was switched to a differentiation medium (DM) consisting of DMEM with 2% horse serum, L-glutamine, and P/S. The timepoints analyzed were days 1, 3, and 6 of differentiation. All tissue culture reagents were from Thermo Fisher, Waltham, MA, USA.

### 2.2. Animal Procedures

In vivo experiments of sclerostin characterization took place in the conventional animal facility of Bordeaux University (agreement number 063-917 obtained 7 May 2018). Eighteen male C57BL/6 mice at the age of 6 weeks (young; *n* = 6), 5 months (adult; *n* = 6) and 18 months (old; *n* = 6) were purchased from Janvier Labs (Saint-Berthevin, France). Animal housing and caretaking were provided by the animal facility in accordance with the national guidelines. All animal procedures were performed in accordance with the European Guidelines for Care and Use of Laboratory Animals.

Mice were kept under a 12 h–12 h light/dark cycle and the temperature was controlled. After the first week of adaptation, animals were euthanized by cervical dislocation and the following muscles were dissected: Gastrocnemius, Soleus, spinotrapezius, and triceps brachii.

Each selected muscle and its contralateral were withdrawn: one was snap-frozen for molecular biology assays and the other was fixed in 4% paraformaldehyde (PFA) for histology.

### 2.3. In Vivo Gene Transfer and Electroporation Procedures

Eight male C57BL/6 mice (Charles River) all belonging to the same progeny were housed in the institutional animal facility (Department of Cellular, Computational and Integrative Biology (CIBIO), University of Trento, Trento, Italy) and maintained on standard chow ad libitum. Three animals (*n* = 3) were electroporated with the SOST vector, three (*n* = 3)—with the empty vector, and two (*n* = 2) were non-electroporated (CTRs). The pCMV6 expression plasmid (MR222588, Origene, Rockville, MD, USA) containing the *SOST* gene tagged with Myc-DDK was amplified in *Escherichia coli*.

Eleven days after birth, mice were anesthetized by inhaled isoflurane and plasmid DNA was injected into the tibialis anterior (TA), quadriceps femoris (QF), and gastrocnemius (GA) muscles with a 0.3 mL insulin syringe through a 31-gauge needle in a constant volume of ~40 μL. Each animal received ~240 γ of plasmid DNA (80 γ/site). A pair of stainless steel plates was then applied over the muscle to encompass the injection area. In all the cases, the distance between the electrodes was 4–4.5 mm. Current was delivered 5 min after DNA injection as a constant current, square wave pulse with a digital stimulator (Panlab 3100, Biological Instruments, Harvard Apparatus, Holliston, MA, USA). The characteristics of the electric field applied were 200 V/cm, 20 ms amplitude, 1 Hz, eight consecutive pulses. The animals were then kept warm until recovery.

The electroporations were performed at days 11 and 17 of age; the animals were then sacrificed at 25 days of age. At each timepoint, animal sera were collected.

Shortly after sacrifice, the animals were X-ray scanned using an In-Vivo Xtreme-Bruker instrument. Animal studies were approved by the institutional Animal Use and Welfare Committee and the National Ministry of Health (protocol No. 62/2020-PR, granted on the 29 January 2020).

### 2.4. Western Blot

At the scheduled timepoints, the cells were lysed with the lysis buffer (10 mM Tris-Cl, pH 7.4–7.6, 1% NP-40, 150 mM NaCl, 10% glycerol, 10 mM EDTA, 20 mM NaF, 5 mM sodium pyrophosphate, 1 mM Na3VO4, and freshly added protease inhibitor cocktail) at 4 °C for 20 min.

Proteins from animal tissues were extracted by addition of the AT buffer sonicated 3 times for 10 s each and incubated for 4 h (muscle) or 24 h (bone) at 4 °C with agitation.

Proteins were resolved by SDS-PAGE, then transferred to 0.2 μm nitrocellulose membranes and probed with the following specific primary antibodies: sclerostin (ABCAM ab63097), tubulin (Sigma Aldrich, St. Louis, MO, USA, T5168), GAPDH (SIGMA G9545). Then, the membranes were washed with Tris-buffered saline Tween (TBST) three times and incubated with specific HRP-conjugated secondary antibodies. Finally, the membranes were treated with enhanced chemiluminescence (ECL) reagents (Bio-Rad, Hercules, CA, USA) according to the manufacturer’s instructions. Images were visualized using Image Lab^TM^ Software (Bio-Rad, Hercules, CA, USA).

### 2.5. RNA Extraction and Quantitative Real-Time PCR

Total RNA was extracted with an RNeasy kit (Qiagen, Hilden, Germany) or TRIzol (Thermo Fisher Scientific, Waltham, MA, USA) and quantified by spectrophotometry with a NanoDrop 2000 device (Thermo Fisher Scientific, Waltham, MA, USA). Then, 500 ng of the total RNA were reverse-transcribed using an iScript^TM^ cDNA Synthesis Kit (Bio-Rad, Hercules, CA, USA). Levels of mRNA were quantitatively determined on a CFX96 Touch^TM^ Real-Time PCR Detection System using an SsoFast™ EvaGreen Supermix according to the manufacturer’s instructions (Bio-Rad, Hercules, CA, USA). PCR primer sequences were as follows: Runx2 forward primer 5′-TTAATCCACAAGGACAGA-3′, Runx2 reverse primer 3′-GTAAGACTGGTCATAGGA-5′; Wisp2 forward primer 5′-TGTGACCAGGCAGTGATG-3′, Wisp2 reverse primer 5′-AGTGACAAGGGCAGAAAGT-3′; Hprt forward primer 5′-GGCATTGCTCTCTCAATGACAA-3′, Hprt reverse primer 5′-ATGTAGGCCATGAGGTCCAC-3′.

For the “In vivo SOST overexpression” experiment (electroporation procedure), the animal tissues (muscles) were probed with sclerostin forward primer 5′-AACAACCAGACCATGAAC-3′, sclerostin reverse primer 5′-TACTCGGACACATCTTTG-3′ (to detect the endogenous sclerostin); sclerostin Myc-Tag forward primer 5′-CGGAGCTGGAGAACGCCT-3′, sclerostin Myc-Tag reverse primer 5′-AATCCAGGATATCATTTGCTGCC-3′ (to detect the exogenous Myc-tagged sclerostin); Hprt forward primer 5′-TCA GAC CGC TTT TTG CCG CGA-3′, Hprt reverse primer 5′-ATC GCT AAT CAC GAC GCT GGG AC-3′.

Relative gene expression values were calculated by applying the 2−∆∆Ct method [26]: using this method, we obtained the fold changes in gene expression normalized to the internal control gene Hprt.

### 2.6. Alizarin Red Staining and Mineralization

Matrix mineralization was evaluated by Alizarin Red S (ARS) staining. The cells were fixed in 4% paraformaldehyde for 20 min at room temperature. Next, the cell layer was stained with 40 mM Alizarin S (Sigma Aldrich, St. Louis, MO, USA - A5533) at pH ~ 4.2 for 5 min under gentle agitation. Cell preparations were washed with PBS to eliminate non-specific staining. To assess the Alizarin Red S staining, 10% cetylpyridinium chloride was added into each well and the cells were incubated for 20 min. Optical density was measured by spectrophotometry at 570 nm.

### 2.7. Alkaline Phosphatase (ALP) Assay

Alkaline phosphatase (ALP) activity was measured with a Alkaline Phosphatase Assay Kit (colorimetric) (Abcam). The staining procedures were performed according to the manufacturer’s suggested protocols. The ALP activity was assessed using p-nitrophenol phosphate (pNPP) as the substrate at pH 10.2 by evaluating the optical density of the yellow substance at 405 nm using an iMark^TM^ Microplate reader (Bio-Rad, Hercules, CA, USA).

### 2.8. Enzyme-Linked Immunosorbent Assay (ELISA)

Sclerostin levels in blood serum samples, animal tissues, and cell lysates were determined with a quantitative sandwich ELISA. Mouse SOST (EM1815, FineTest, Wuhan, Hubei, China) ELISA kits were used according to the standard protocols provided by the manufacturer. All samples were assayed in duplicate.

### 2.9. Immunofluorescence

C2C12 and C57 cells grown on glass coverslips were fixed in 4% paraformaldehyde at 4 °C for 20 min, blocked in 4% (*w/v*) bovine serum albumin (BSA) PBS and incubated with primary anti-sclerostin (ab63097, Abcam, Cambridge, United Kingdom) anti-MyoD antibody (Santa Cruz Biotechnology, Dallas, USA) overnight at 4 °C. Coverslips were next washed with PBS and incubated with fluorescence-labeled secondary antibodies (Thermo Scientific, Waltham, MA, USA) for 1 h at room temperature. Slides were mounted with a 10% DABCO (1,4-diazabicyclo[2.2.2]octane) solution and were observed using a Nikon A1 confocal laser scanning microscope. The confocal serial sections were processed with ImageJ software to obtain three-dimensional projections and image rendering was performed using Adobe Photoshop CS 8.0 software (Adobe Systems, San Jose, CA, USA). All the images shown in this paper are representative of at least 3 independent experiments carried out under the same conditions.

### 2.10. Histological Staining and Immunohistochemistry

Animal tissues (muscle, bone) were fixed with 4% PFA overnight. Next, the samples were processed for paraffin embedding (Leica EG1150 H) following the procedure described here: 70% ethanol for 30 min, three times in 95% ethanol (1 h each), three times in 100% ethanol (1 h each), three times in toluene (1 h each), and two times in paraffin (1 h each). After paraffin embedding, 5–7 μm sections were obtained from each sample by means of a Leica RM2155 microtome (Leica Inc. Wetzlar, Germany).

Hematoxylin and eosin (H&E) staining was performed on murine muscles and bone tissues. Briefly, two slides per muscle were deparaffinized by means of xylene (two times for 5 min each), hydrated through a graded series of alcohol (100%, 95%, 80%, and 70%) for 5 min each, and rinsed; first with tap water and then with deionized water for 1 min each. After the dehydration procedure, the slides were stained with a Carazzi hematoxylin solution (C0203, DiaPath, Bergamo, Italy), rinsed with tap water, stained with alcoholic 0.5% eosin Y (C0353, DiaPath, Bergamo, Italy), and then rinsed with deionized water for 1 min each.

Histomorphometric analysis was conducted on the right and left femurs. Briefly, both femurs were removed from each animal, deprived of soft tissues, fixed in PBS—4% paraformaldehyde (pH 7.4), dehydrated in graded ethanol, and paraffin-embedded. The femurs were cut sagittally at the distal metaphyseal level and transversally cut at the mid-diaphyseal region to obtain serial 5 μm-thick sections for histomorphometric analysis (ImageJ software, 1.52v, NIH). The sections were stained with H&E to measure the following static histomorphometric parameters: trabecular bone volume (BV/TV), trabecular thickness (Tb.Th), trabecular number (Tb.N), and trabecular separation (Tb.Sp) of the distal femoral metaphyses, as well as the cortical bone area (Ct-B-Ar) of femoral mid-diaphyses. The areas in which the measurements were performed were selected as previously described.

For immunohistochemistry staining, from the paraffin block of each case, we cut a 4 µm-thick slides for immunohistochemical analysis against CD34 (pre-diluted QBEnd/10 Mouse Monoclonal Antibody clone; Ventana Medical Systems, Tucson, AZ, USA) and CD45/LCA (pre-diluted 2B11&PD7/26 Mouse Monoclonal Antibody clone; Cell Marque™- a Sigma-Aldrich^®^, St. Louis, MO, USA) using a Bench Mark ULTRA automated stainer (Ventana Medical Systems, Tucson, AZ, USA).

For both antibodies, microwave oven irradiation was used as the immunohistochemical antigen unmasking treatment. In addition, a pre-diluted ULTRA Cell Conditioning Solution (ULTRA CC1—Ventana) was used as a pre-treatment step in the processing of tissue samples for immunohistochemistry reaction. Sample sections of the vascular tumor and lymphoid tissue were used as the internal control for CD34 and LCA/CD45 markers, respectively. The sections were blocked for non-specific binding with 3% normal bovine serum and incubated with primary antibodies for 1 h at room temperature. For immunofluorescence, the sections were then incubated with secondary antibodies at 1:200 for 30 min at room temperature (DABCO). The sections were mounted with a VECTASHIELD HardSet mounting medium. All the stained sections were evaluated under a Nikon Eclipse Ni microscope (Nikon) equipped with a DS-Fi2 camera (Nikon) and processed by means of NIS-Elements D 5.11.00 software (Nikon, Rochester, New York, NY, USA).

### 2.11. Micro-CT Measurements

Micro-CT scanning was carried out on both left and right tibiae of C57BL/6 mice after electroporation and bone harvesting. Micro-CT scanning and analysis were performed using a KEVEX PXS10 130 kV–0.5 mA X-ray tube and a Photonic Science VHR1 CCD (charge-coupled device) camera with a fiber optic plate with a scintillator (FOS) 4000 × 2600 (9 μm pixel). The isometric voxel size was 4.5 μm and the X-ray tube potential was 70 kV.

For each tibia, axial sections (from distal epiphysis to middle diaphysis) were acquired and stacked in a single DICOM (Digital Imaging and Communications in Medicine) file. The acquisition volume was reconstructed from each DICOM file and the coronal sections were then extracted. The most informative (i.e., with less background and more signal, that is, imaged bone) coronal section was selected and analyzed together with the previous five and the subsequent five for a total of 11 coronal sections per sample. For each section, the epiphysis, metaphysis and metaphyseal plate were segmented separately and automatically; in each of the 11 sections, the metaphysis was further segmented through the subdivision of metaphyseal trabeculae into 16 different types, or classes. To this purpose, a Gabor filter bank was used, as it is a well-established approach to analyze objects’ thickness and orientation in grey-level images in computer vision applications [27]. In the case of our classification, the trabecular thickness can have a value of 2 or 3 pixels (therefore, it is related to a range of about 10–15 µm), while the angle of trabecular inclination with respect to the metaphyseal plate w quantized by multiples of 22.5 degrees. Thus, the classes of metaphyseal trabeculae were defined by a unique combination of trabecular thickness and orientation as follows in Table 1.

Each trabecular class identified was analyzed separately considering the trabecular number, thickness, shape, and area. The statistical analysis was performed pooling the left and right tibiae of the same experimental group (untreated (CTR), empty vector-treated (EV-ET), and sclerostin vector-treated (SOST-EV)). First, the three groups of tibiae were compared to each other in pairs (i.e., CTR against EV-ET, CTR against SOST-ET, and EV-ET against SOST-ET, separately). Then, for each pair of groups, their individual tibiae were compared in pairs by considering all the possible pairs of inter-group comparison in order to verify whether what is observed from the comparison between the groups can also be valid for the individual tibiae that compose them. As an example, if the CTR group is composed of tibiae 1A and 1B and the EV-ET group is composed of tibiae 2A and 2B, after pair-comparing the whole groups (CTR vs. EV-ET), the single samples that compose them are pair-compared in all possible combinations (1A vs. 2A, 1A vs. 2B, 1B vs. 2A, 1B vs. 2B).

### 2.12. Statistical Analyses

All the data were analyzed using Prism version 6.0e (GraphPad Software, Graph Pad Holdings, CA, USA). In vitro data are presented as the means ± standard deviation (SD) from three independent experiments. Unless otherwise stated, the data for animal experiments are expressed as the means ± standard deviation, and statistical analyses were performed using one-way ANOVA followed by Dunnett’s, Sidak, or Tukey’s multiple comparison tests. Differences between groups were considered statistically significant at *p* < 0.05.

## 3. Results

### 3.1. Analysis of the Effects Mediated by Myogenic Medium on Osteoblast Differentiation

**Figure 1:**
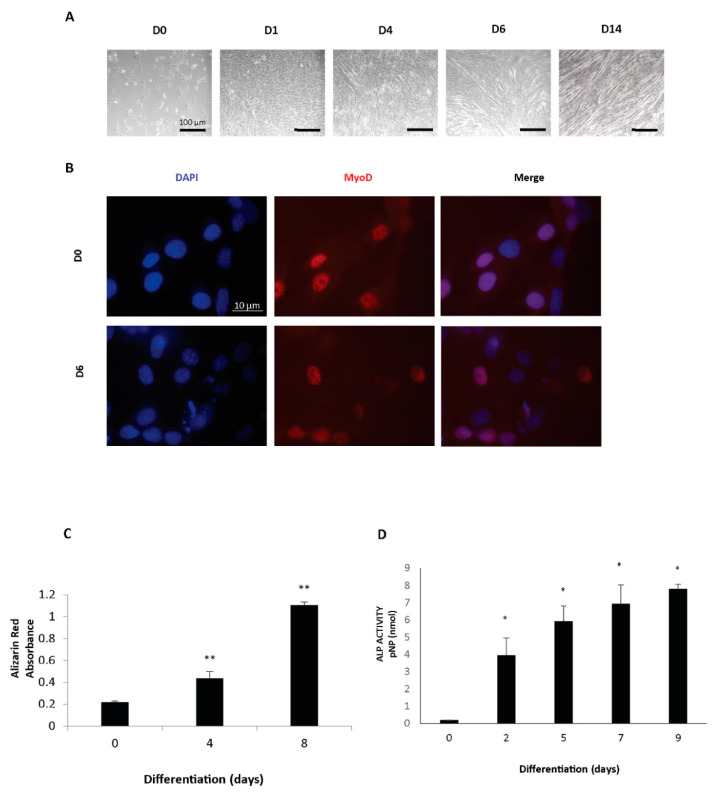
(**A**) Representative bright-field images of C2C12 cells along the differentiation process. D0: cycling cells. D1, D4, D6, D14 are differentiated cells for 1, 4, 6, and 14 days, respectively. (**B**) Immunofluorescence staining of MyoD of C2C12 cycling cells (D0) and differentiated cells (D6). Blue color is DAPI staining and Red color is MyoD staining (**C**,**D**) Alizarin Red staining (**C**) and ALP assay (**D**) of 2T3 cells differentiated for the indicated days. Student’s *t*-test (vs. the sample named 0) was applied with *p* < 0.05 (*) and *p* < 0.005 (**).

In the first part of this study, we proposed modelling the muscle-to-bone crosstalk by differentiating 2T3 osteoblastic cells in a medium conditioned by C2C12 muscle cells. Particularly, to investigate the effect of sequential patterns of myokines secreted during the process of muscle differentiation, we planned to use media from C2C12 cultures maintained in differentiating conditions for different amounts of time. With the goal to set the proper conditions for this experiment, we initially characterized the differentiation process in both myogenic C2C12 cells and osteogenic 2T3 cells lines. Figure 1A illustrates the morphological changes occurring during the in vitro lineage progression of C2C12 myoblasts. Terminally differentiated myotubes were initially apparent after four days of culture and became progressively predominant at later timepoints. The myogenic properties of the C2C12 cells were confirmed by the presence of the well-known myogenic transcription factor MyoD (Figure 1B). In order to evaluate differentiation of the osteoblast cell line 2T3, we performed functional assays, such as the Alizarin Red assay and the Alkaline Phosphatase Assay, demonstrating a progressive increase in the mineral matrix amount and enzyme activity during the osteogenic maturation process (Figure 1C,D).

**Figure 2:**
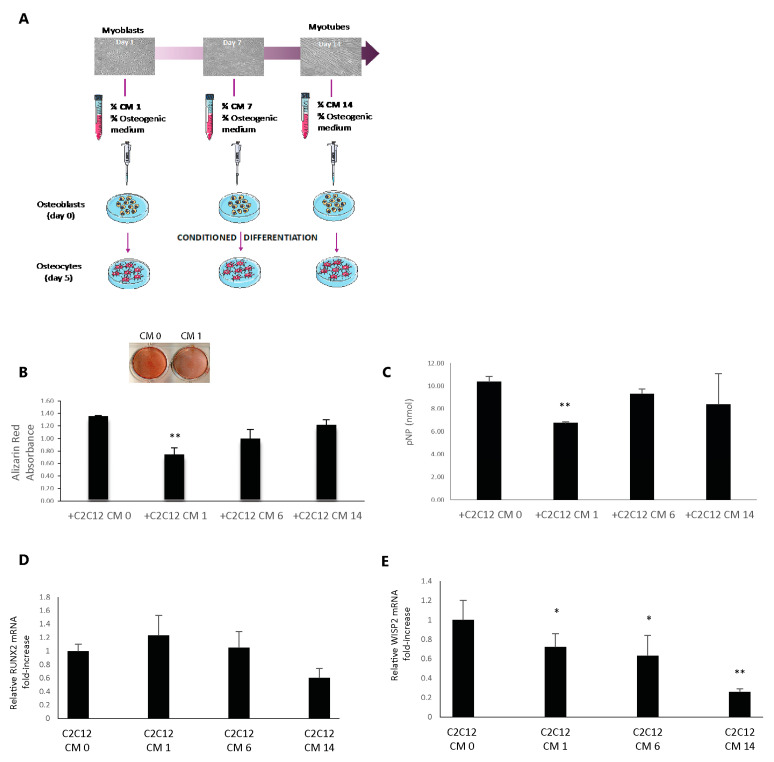
(**A**) Graphical scheme of the experimental workflow. A quarter of the medium obtained by C2C12 cells in cycling (CM0 used as reference) and in differentiating conditions (CM1–CM7–CM14) was added to three quarters of the osteogenic differentiating medium on 2T3 cells. Osteoblast cells were maintained in the differentiation medium for five days. (**B**,**C**) Alizarin Red staining and ALP assay of differentiated 2T3 cells conditioned with C2C12 media (CM0–CM1–CM6–CM14). Student’s *t*-test (vs. sample CM0) was applied with *p* < 0.05 (*) and *p* < 0.005 (**) (**D**,**E**) Messenger RNA was extracted from differentiated 2T3 cells and the relative expression of the osteogenic markers Runx2 and Wisp2 was evaluated. Statistical significance was obtained using one-way ANOVA + Dunnett’s post test vs. CM0.

After having established a protocol for efficient differentiation of C2C12 myoblasts and 2T3 osteoblasts, we collected C2C12 culture supernatants at early (1 day of culture in differentiating conditions, CM1), mid (6 days of culture, CM6), or late (14 days of culture, CM14) stages of differentiation and we tested their effects on osteoblast maturation by adding 25% C2C12-conditioned media to the osteogenic differentiation medium along 5 days of culture (Figure 2A). We set up a control condition by adding a 25% cycling myoblast medium to the osteoblast maturation medium (CM0). Alizarin staining revealed a significant decrease in mineralization (i.e., maturation) of the osteoblasts differentiated with CM1*** compared to the control condition (CM0) (Figure 2B). Furthermore, the CM6 condition and, at a minor extent, the CM14 condition showed a tendency toward reduced mineralization. The ALP activity assay revealed a similar trend showing the CM1 condition significantly lower than the CM0 controls (Figure 2C). Therefore, our initial observations indicate that the factors dynamically secreted by differentiating C2C12 cells are able to significantly alter the functional maturation of osteoblasts.

Next, we attempted to further characterize the impact of the C2C12-conditioned media on the 2T3 osteogenic program by performing an RT-PCR analysis of the osteogenic genes *Runx2* and *Wisp2*, an endpoint of the Wnt signaling pathway that was recently described as a reliable marker of osteogenesis by our group [28]. While we found a constant expression of Runx2 during myogenic modulation of the 2T3 osteoblast differentiation (Figure 2D), the transcription level of Wisp2 was consistently reduced, with statistically significant differences during myogenic conditioning from the CM1 to the CM14 setpoint (Figure 2E), corroborating the data obtained through the functional Alizarin and ALP assays.

### 3.2. Sclerostin Characterization in Muscle Cell Lines and in Primary Muscle Tissues

With the goal to get mechanistic insights into the agent putatively responsible for the slowdown of osteogenic maturation induced by muscle cells, we pointed our attention to negative modulators of bone mineralization that are reportedly able to modify the Wnt signaling pathway and therefore may explain the dramatic alteration of Wisp2 expression induced by the muscle-conditioned media. Particularly, we hypothesized that a possible candidate could be sclerostin. Indeed, sclerostin is a major secreted inhibitor of bone formation and stands out as an important actor upstream of the Wnt signaling pathway. Sclerostin was generally considered an osteocyte-specific protein, since sclerostin mRNA and proteins were detected in mouse and human bones, and the expression was restricted to osteocytes [23,24]. Despite circumstantial documentation of the presence of *SOST* transcripts in other organs, including skeletal muscles, little attention has been given so far to other tissues as a potential source of sclerostin [29].

**Figure 3:**
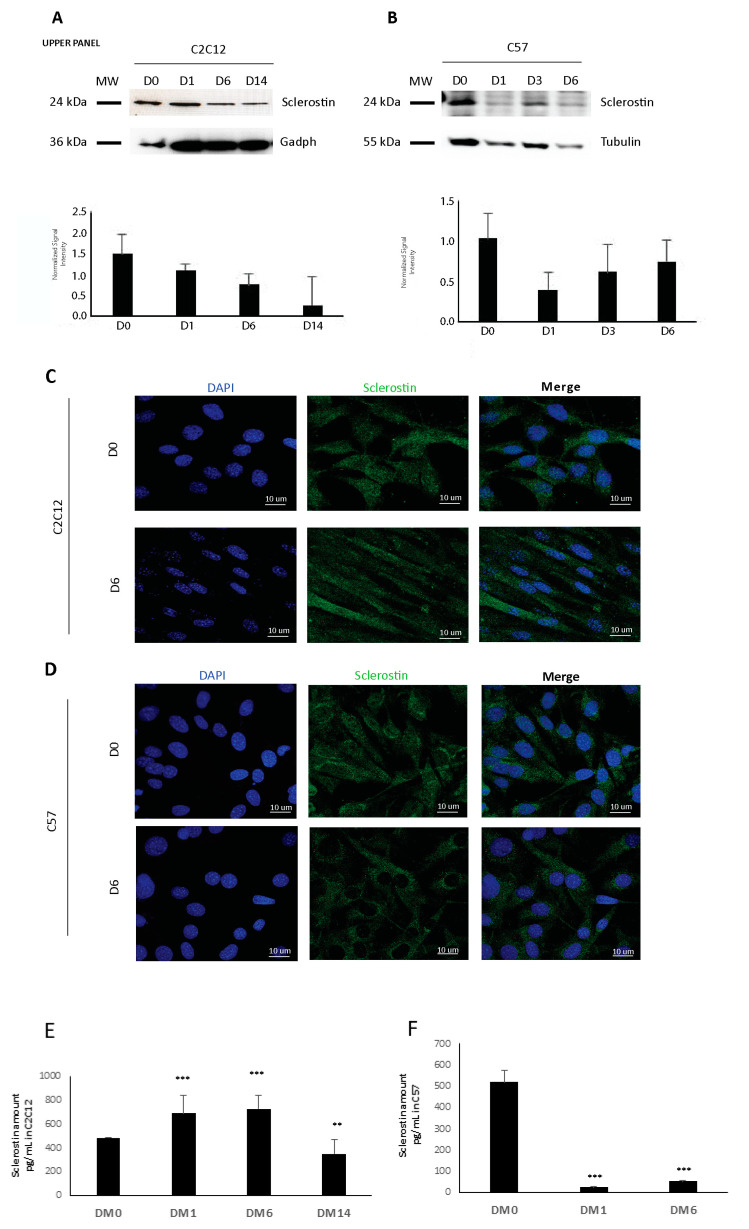
(**A**,**B**) C2C12 and C57 maintained in the cycling (DO) and differentiating conditions (D1–D3–D6–D14) were analyzed by Western blotting (upper panel). Forty ug of protein extracts were resolved by SDS-PAGE and sclerostin expression was revealed. GAPDH/actin or tubulin antibodies were used to normalize the signal intensity for at least three independent experiments (lower panel). (**C**,**D**) Immunofluorescence analysis of sclerostin in C2C12 and C57 cells in the growing (D0) and differentiating conditions (D6). (**E**,**F**) Quantitative analysis through ELISA assay of the sclerostin secreted in the media produced respectively by C2C12 and C57 cells in the cycling and differentiating conditions. Student’s *t*-test (vs. sample named DM0) was applied with *p* < 0.005 (**) *p* < 0.0005 (***). The experiment was repeated in triplicate.

Therefore, we supposed that it may be involved in the effects shown in the muscle cells-conditioned medium during osteogenesis and we decided to investigate for the first time the potential synthesis and secretion of sclerostin by muscle cells. Interestingly, we found the presence of the sclerostin protein both in C2C12 cells and in the primary myoblasts obtained from the muscles of C57 mice. Western blotting revealed that sclerostin is readily detectable in cellular extracts of cycling myoblasts and differentiating cells (Figure 3A,B). Although differences in intracellular levels of the sclerostin protein were not statistically significant between the considered timepoints, these data clearly demonstrate the ability of myogenic cells to synthetize the sclerostin protein. The expression of sclerostin was also confirmed by immunofluorescence that showed its localization in the cytoplasm according to its nature of a secreted glycoprotein (Figure 3C, D). Importantly, we also quantified with the ELISA assay the amount of sclerostin released by C2C12 and C57 cells into the media (Figure 3E,F). Altogether, this analysis demonstrated that sclerostin is expressed by C2C12 and C57 cells at the protein level in early, mid, and late stages of differentiation and that it is secreted in the medium in a dynamic way during myogenic lineage progression.

**Figure 4:**
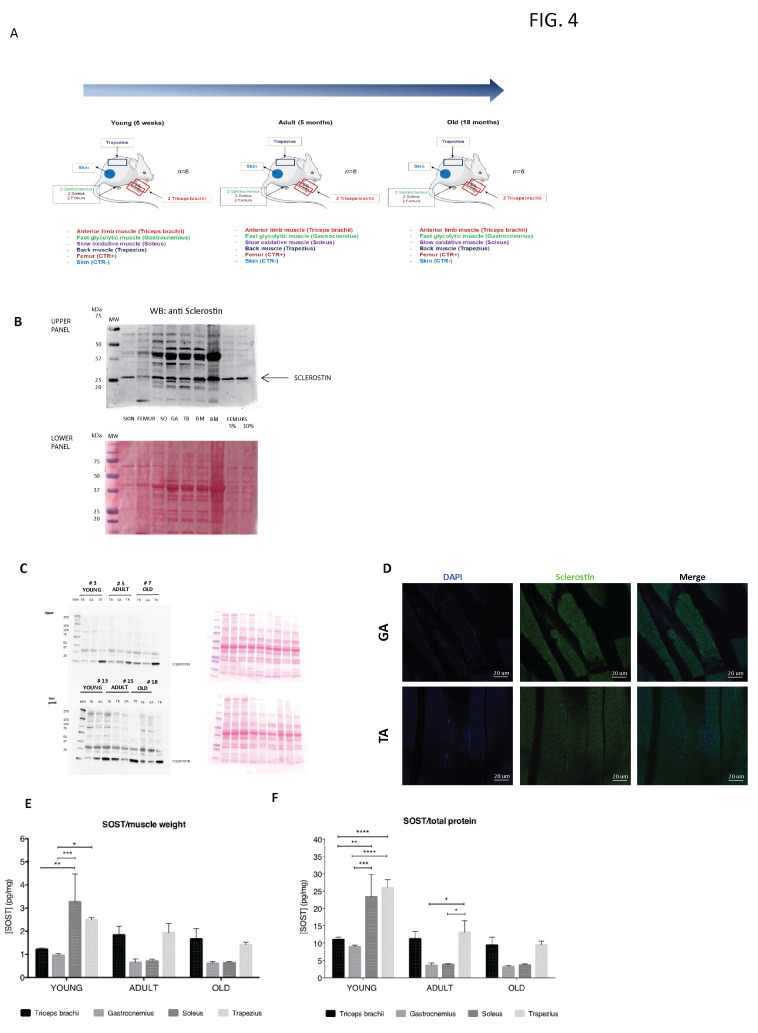
(**A**) Graphical scheme of the muscles obtained in mice of different ages. (**B**) Twenty ug of protein extracts obtained in different tissues were analyzed for the expression of sclerostin under normal and higher (5–10% β-mercapthoetanol) reducing conditions (upper panel). Ponceau staining for visualization of the equal number of loaded samples is in the lower panel. (**C**) Sclerostin expression in protein extracts obtained in different muscles. For each group of muscles, the sclerostin expression analysis was repeated for 2 animals (upper panel). Ponceau staining for visualization of the equal amount of loaded samples is represented in the lower panel. (**D**) Frozen sections of the gastrocnemius muscle were subjected to immunofluorescence staining of sclerostin. The cell nuclei were stained with DAPI. Scale: 20 µm. (**E**,**F**) ELISA assay measuring the sclerostin amount in protein extracts from distinct muscle groups collected in mice of different ages: young (6 weeks), adult (5 months), old (18 months). The data are represented as the sclerostin amount related to muscle weight (**E**) and to the total extracted protein (**F**). * *p* < 0.05, ** *p* < 0.005, *** *p* < 0.0005, **** *p* < 0.0001.

To corroborate our observation of the production of sclerostin by muscle cells in vitro, we moved to the in vivo context by analyzing the presence of sclerostin in muscles by Western blotting, immunofluorescence, and ELISA techniques. The analysis was performed on muscles of mice of different ages (young, 6 weeks; adult, 5 months; old, 18 months). For each age, muscles with different metabolic profiles were analyzed in parallel. Specifically, to observe eventual relations between muscular work and sclerostin production, we isolated (i) a muscle predominantly composed of fast or glycolytic fibers (gastrocnemius, GA); (ii) a muscle containing a high fraction of slow oxidative fibers (soleus, SO), which are subjected to phasic movement and important muscular work; (iii) a muscle of the back (trapezius, TR), less loaded according to the phasic activity of the limbs. Furthermore, a muscle of the fore limbs (triceps brachii, TB) was isolated to compare it with the muscles of the hind limbs (Figure 4A).

First, to ensure proper semi-quantification of sclerostin by Western blotting, we decided to verify the specificity of the bands recognized by the sclerostin antibody under different experimental conditions. To this aim, we processed different mouse tissues such as skin and a number of muscle types and we compared the resulting pattern of bands with the pattern obtained with femur extracts homogenized in the normal lysis buffer or in a lysis buffer with higher reducing conditions (respectively, 5% and 10% of β-mercapthoetanol). The Western blot image shown in Figure 4B highlights the presence of a “bona fide” specific band clearly visible in the femur protein extracts under higher reducing conditions. These results suggest that the sclerostin antibody is suitable for the Western blot analysis despite the presence of an additional aspecific band migrating slower compared to the specific one, particularly apparent in the tissues homogenized with the normal lysis buffer. Next, we employed the Western blot technique to analyze the expression of sclerostin in distinct muscles obtained from mice of different ages (Figure 4C). Two animals per age group were probed. The results obtained with Western blotting show that sclerostin is synthesized by all the muscles analyzed with a specific band at the expected molecular weight. The presence of the sclerostin protein in the muscle fiber cytosol was also confirmed by immunofluorescence on cryosections of gastrocnemius and tibialis anterior muscles (Figure 4D).

The results of the Western blot analysis also suggest that sclerostin expression might be heterogeneously expressed by different muscle groups. By employing a quantitative approach such as the ELISA assay, we confirmed the trend revealed by Western blotting. The sclerostin quantification obtained by the ELISA assay after normalization for both muscle weight and the total protein content indicates that muscular sclerostin production is dynamically modulated with time in a muscle group-specific way (Figure 4E,F).

### 3.3. In Vivo Muscle-Specific Overexpression of Sclerostin

**Figure 5:**
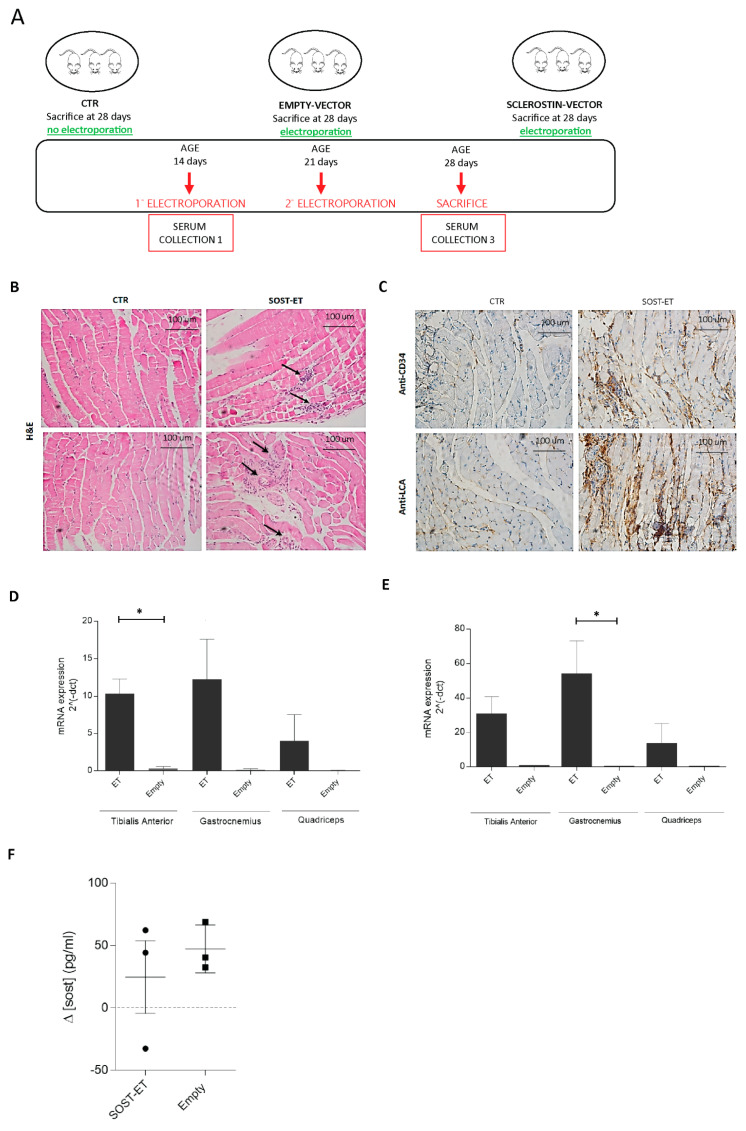
(**A**) Graphical scheme of the sclerostin overexpression experiment. Mice were randomly assigned to three different experimental groups: non-electroporated control (CTR) or electroporated with an empty vector or with the sclerostin vector. At the baseline time (before the first electroporation) and before sacrifice, the serum was collected from each animal. (**B**,**C**) The photographs show representative histological sections of the tibialis anterior of C57BL/6 mice. Magnification 20X. Sections of CTR and SOST-ET (SOST-electroporated) mice stained with H&E. The black arrows indicate inflammatory infiltrate (**B**). Sections stained with anti-CD34 and anti-LCA antibodies (**C**). (**D**,**E**) Exogenous myc-tagged (**D**) and total sclerostin (**E**) expression were evaluated by RT-PCR in tibialis anterior, gastrocnemius, and quadriceps femoris muscles electroporated with the SOST plasmid (ET, *n* = 3) or electroporated with an empty vector (Empty, *n* = 3, except for tibialis anterior, *n* = 2). Data are expressed as the means with the SEM. For each muscle, statistical analyses were performed using an unpaired *t*-test (two-tailed). * *p* < 0.05. (**F**) Serum sclerostin levels evaluated through ELISA and calculated as the delta (Δ) amount of sclerostin before the electroporation as a starting level and at the moment of sacrifice.

In order to investigate a possible role for the muscle form of sclerostin, we performed a gain-of-function experiment. We overexpressed transiently, via electroporation (ET), a plasmid containing the *SOST* gene in muscles of young mice. As detailed in Figure 5A, this study was based on 3 experimental groups: control (CTR, 2 mice sacrificed at 25 days of age without electroporation); empty vector (EV-ET, 3 mice electroporated with the pCMV6 vector) and sclerostin vector (SOST-ET, 3 mice electroporated with the pCMV6 vector in which the *SOST* gene was cloned). The electroporation was first performed at 11 days of age and repeated 6 days later, since the exogenous DNA is stable for nearly 7 days [29]. Animals were sacrificed at 25 days of age. The muscles selected were quadriceps femoris (QF), gastrocnemius (GA), and tibialis anterior (TA) of both hind limbs, which allowed targeting the skeletal segments of femur and tibia with the paracrine secretion of sclerostin.

First, to assess the potential injury induced by electroporation, we performed histological analysis of the muscles subjected to the procedure. Clinical symptoms were not observed in any of the mice during the observation period of two weeks. At sacrifice, the electroporated muscles were stained with hematoxylin and eosin (H&E) and with anti-CD34 and anti-LCA antibodies (Figure 5B,C). Microscopic examination of H&E-stained fibers in SOST-electroporated mice revealed a limited presence of inflammatory infiltrates (Figure 5B, black arrows), whereas control samples present fibers correctly arranged with subsarcolemmal (i.e., peripheral) nuclei. Moreover, the immunohistochemical localization of the human leukocyte common antigen (LCA) (Figure 5C), a major membrane glycoprotein restricted to leukocytes, showed a recall of granulocytes. Despite the induction of inflammation, newly formed vessels were visible in electroporated mice (Figure 5B, white arrows), suggesting a local activation of the regenerative process. Indeed, functional vascularization was confirmed by the staining with anti-CD34, detecting a transmembrane phosphoglycoprotein expressed almost ubiquitously by hematopoietic cells. Electroporated muscles presented positively-stained regions at the level of muscle mass and the surrounding connective tissue (Figure 5C). Taken together, these data suggest that the electroporation procedure may induce mild inflammatory reactions, which are counterbalanced by the onset of regenerative processes.

Next, we checked the electroporation efficiency. We performed a quantitative reverse transcription-PCR (qRT-PCR, Figure 5D,E) analysis to detect both the myc-tagged sclerostin mRNAs transcribed from the plasmid containing the exogenous *SOST* gene and the total sclerostin mRNAs, which includes the endogenous and exogenous transcripts. The results showed consistently a level of myc-tagged sclerostin transcripts in all the muscles electroporated with the plasmid containing the *SOST* gene compared to the ones injected with the empty vector; this comparison reached statistical significance (*p* < 0.05) in tibialis anterior (TA) muscles. This result was confirmed by the analysis of total sclerostin expression, which reached significance (*p* < 0.01) in GA muscles.

Although electroporation was performed on a specific set of hind limb muscles (TA, GA, QF) which represent only a limited fraction of the entire body muscle mass, we decided that evaluating SOST plasmid electroporation might be sufficient to alter the quantity of circulating sclerostin. To this aim, we quantified the presence of sclerostin with the ELISA assay in animal sera before the first electroporation as a starting level and at the moment of sacrifice (Figure 5D). Although the average physiological concentration of circulating sclerostin was higher in the SOST-electroporated animals compared to the EV-electroporated animals both before electroporation (respectively, 360.7 ± 18.05 pg/mL vs. 273.3 ± 30.3 pg/mL) and at sacrifice (respectively, 385.5 ± 68.5 pg/mL vs. 320.7 ± 22.3 pg/mL), probably due to individual variability between experimental animals, the average amount of circulating sclerostin appeared not to be significantly altered by the electroporation of the SOST-expressing plasmid (Figure 5F). The raw data obtained in these experiments are listed in Table S1.

Taken together, these data confirmed that plasmid electroporation was successful in yielding a strong local increase in sclerostin in the targeted muscles but highlight a limited influence on the circulating levels of sclerostin.

### 3.4. Analysis of SOST Overexpression Effects on the Skeleton

**Figure 6:**
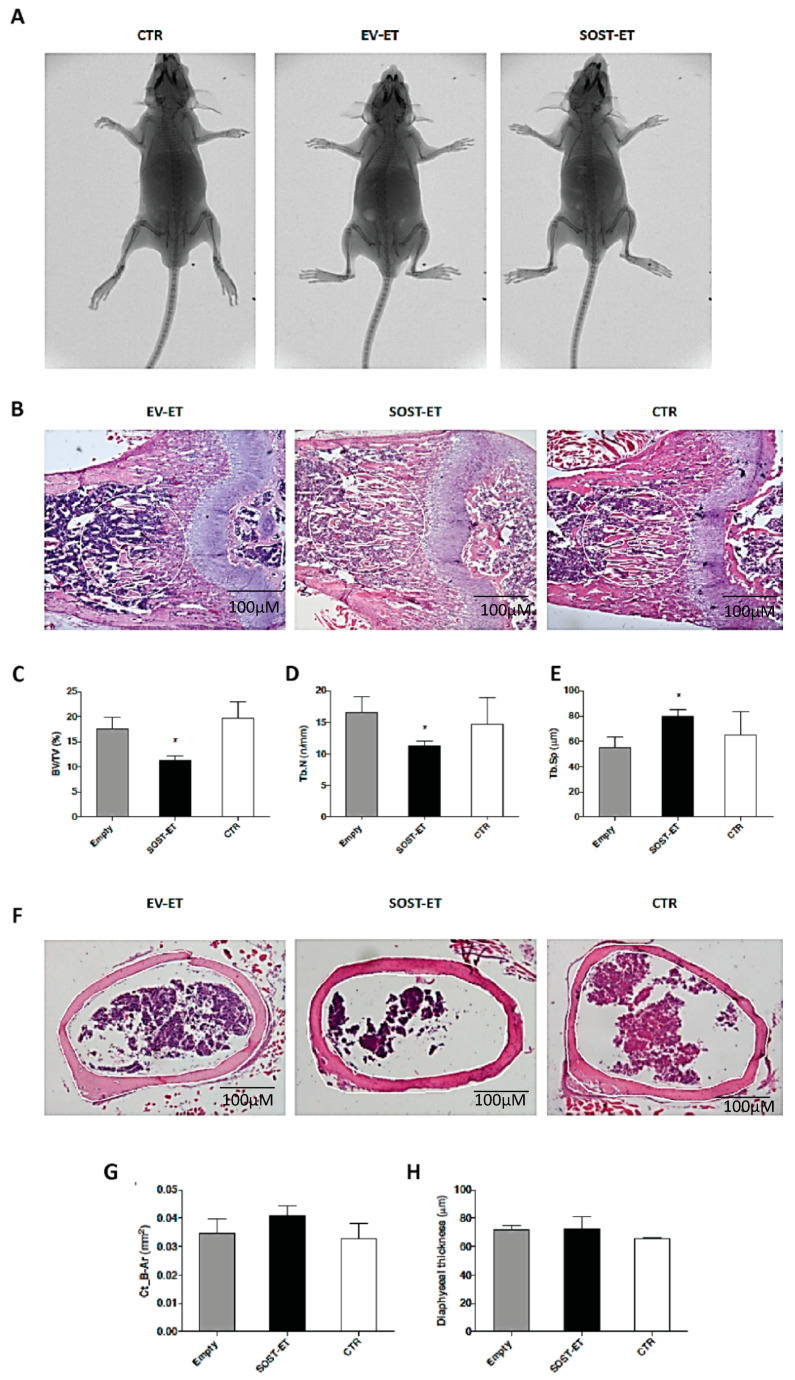
(**A**) Representative X-ray scans of the skeleton of each mice group: CTR, EV-ET, SOST-ET. (**B**) Sagittal section of the distal third of femur (4× magnification). Exemplificative histological sections (5 mm) of femurs of non-electroporated (CTR), empty vector (EV-ET), and SOST-vector electroporated animals in which the histomorphometric analyses were performed. (**C**–**E**) Mean values of histomorphometric parameters expressed as BV/TV (%), Tb.N (n/mm), Tb.Sp (mm) in trabecular bone of the three animal groups (transversal section at the mid-diaphyseal level of femur) (10X magnification). The white line indicates the areas in which histomorphometric evaluations were recorded. (**F**) transversal section at the mid-diaphyseal level of femur (10X magnification). The white line indicates the areas in which histomorphometric evaluations were recorded. (**G**,**H**) Mean values of histomorphometric parameters expressed as CT_B-Ar (mm^2^) and diaphyseal thickness (mm) in cortical bone of the three animal groups. Values are expressed as the means ± SEM. * *p* < 0.05.

In order to evaluate potential systemic effects on skeletal growth exerted by the transient muscular overexpression of sclerostin, we analyzed the responses at the level of SOST-electroporated, EV-electroporated, and control littermate mice using X-ray scans. The following parameters were evaluated: (i) skeletal malformations of individual bone segments, (ii) changes in bone density, (iii) focal structural alterations of bone segments, (iv) alterations of the juxta-osseous soft tissues (calcifications or masses). From the radiographic comparison of the skeleton of the CTR group (non-electroporated) and of the electroporated groups (EV- or SOST-electroporated), it emerged that no growth anomalies were found following the electroporation treatment. Furthermore, we could not observe differences, at least at the macroscopic skeletal level, which can be ascribed to the electroporation of the vector expressing sclerostin (Figure 6A, representative set of radiographic images).

The absence of a generalized bone phenotype is in keeping with inefficient alteration of circulating sclerostin levels exerted by SOST plasmid electroporation (see above). Nevertheless, accumulating evidence indicate that myokines may affect adjacent bones through paracrine mechanisms, relying on their diffusion across muscle and bone tissues [30]. In order to evaluate the microscopic events induced by muscle electroporation on adjacent skeletal segments, we performed histological staining of bone sections of femurs from EV-electroporated (EV-ET), SOST-electroporated (SOST-ET), and non-electroporated control (CTR) animal groups (Figure 6B,F). The bone amount was clearly lower in SOST-ET with respect to EV-ET and CTR animals. This observation was confirmed by the static histomorphometric analysis. The ratio between the trabecular bone area (BV) and the total area (TV) (i.e., the trabecular bone volume, BV/TV), the trabecular number (Tb.N), and the trabecular separation (Tb.Sp) were recorded from the trabecular bone of femur (Figure 6C–E), whereas the cortical bone area (Ct-B-Ar) values and diaphyseal thickness were recorded from femur cortical bone (Figure 6G,H). The results obtained in trabecular bone of the femur distal metaphysis show that the mean values of BV/TV and Tb.N in the SOST-ET group were statistically lower with respect to the EV-ET group. Moreover, in the SOST-ET group, the Tb.Sp value was significantly higher than in the EV-ET group. Conversely, no statistically significant differences were found in the cortical bone area (Ct-B-Ar) and diaphyseal thickness at the femoral mid-diaphyseal level among the three groups. Collectively, these data suggest that the muscular overexpression of sclerostin induced a loss of trabecular bone, but it did not influence cortical bone parameters. These data are in line with the fact that the trabecular bone, compared to the cortical bone, has a higher turnover rate and is more metabolically active than the cortical bone, thus being more easily influenced [31,32].

**Figure 7:**
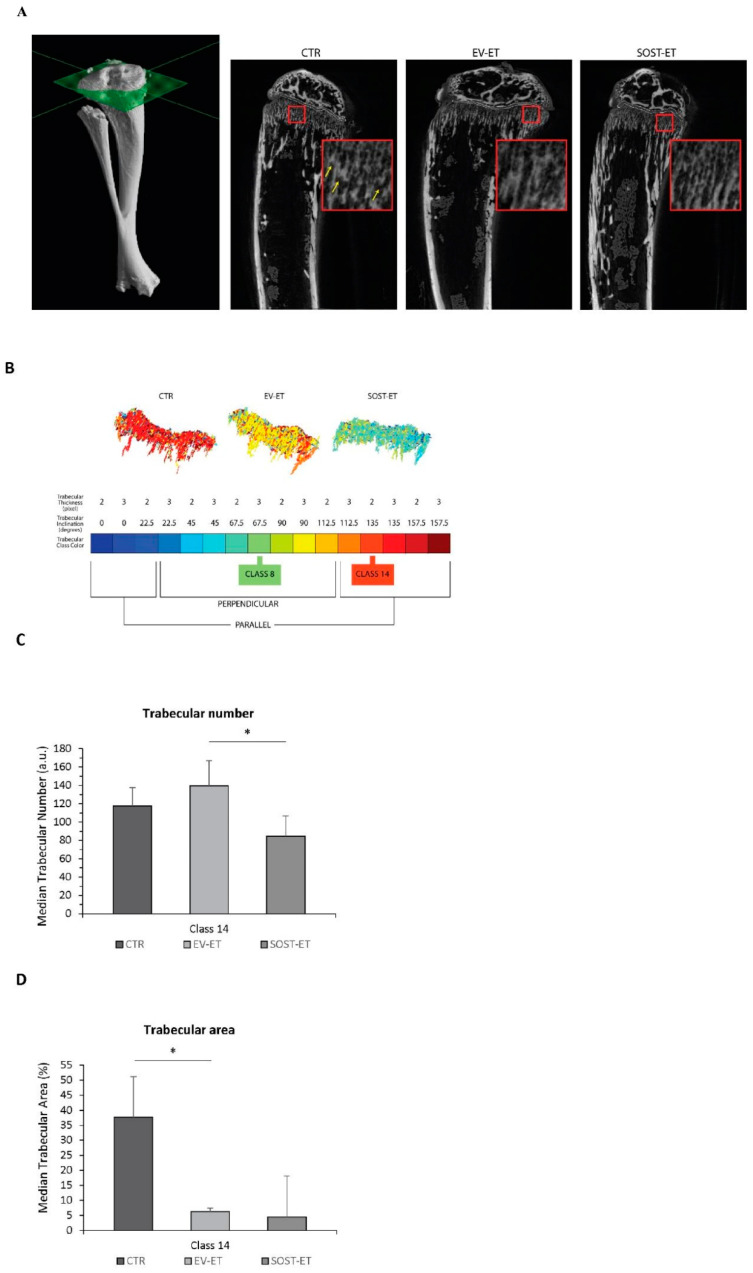
(**A**) Exemplificative µCT scans of the metaphyseal plate for each group of animals: CTR, EV-ET, and SOST-ET. Yellow arrows show the trabecular orientation. (**B**) Heat maps that represent the space occupied by the various classes with a color code. Legend of the major represented trabecular classes. Class 8 (yellow, green) is representative of perpendicular trabeculae and Class 14 (red) is representative of parallel trabeculae. (**C**,**D**) Variation in median values of the trabecular number and trabecular area for trabeculae of class 14 (± median absolute deviation (MAD) of trabeculae for classes (typology)). Asterisk (*) indicates significant comparisons, *p* < 0.05 according to the Wilcoxon–Mann–Whitney test with the Bonferroni correction.

In order to further investigate the effects of muscle-specific sclerostin overproduction in adjacent muscles of growing mice via electroporation of a plasmid containing the *SOST* gene, micro-CT analyses were performed on tibiae, which lie between the TA and gastrocnemius (GA) muscles targeted by the electroporation. For each tibia, axial sections (from distal epiphysis to middle diaphysis) were acquired and then the coronal sections were extracted, on which the analysis of the epiphysis, metaphysis, and the epiphyseal plate were performed. Figure 7A shows exemplificative coronal sections. At the regional level (i.e., epiphysis, metaphysis, epiphyseal plate, and diaphysis as wholes), no macroscopic changes in the trabecular organization were detected either in response to the EV electroporation or in response to SOST electroporation.

The metaphysis was thus further segmented through the subdivision into 16 different classes of metaphyseal trabeculae (see Materials and Methods). A representative false color map of the composition of trabecular classes present in tibiae from EV-electroporated (EV-ET), SOST-electroporated (SOST-ET), and non-electroporated control (CTR) animals is shown in Figure 7B and highlights differences between experimental groups. Analyses were focused on two types of bone trabeculae differently arranged with respect to the metaphyseal growing plate in the longitudinal (class-8, PERP) and parallel (class-14, PAR) mode. Compared to the control group, the number of trabeculae of class 8 (PERP) increased following electroporation with an empty vector (+30%, *p* = 10^−5^, *p* < 0.0021) and then decreased to the levels comparable to those of the control group following electroporation with sclerostin, although not in a significantly manner (data not shown). Compared to the control group, the number of trabeculae of class 14 (PAR) increased following EV electroporation, albeit without significance (EV-ET compared to CTR: +16%, *p* = 0.040, threshold *p* < 0.0021), but decreased significantly following SOST electroporation (SOST-ET compared to EV-ET: −39%, *p* = 10^−10^, threshold *p* < 0.0014) (Figure 7C). The percentage of metaphysis occupied by the trabeculae (trabecular area) of class 8 (PERP) increased significantly (compared to the control group) following electroporation with an empty vector (+63%, *p* = 10^−5^, *p* < 0.0021) and further increased following electroporation with sclerostin (data not shown). As regards the trabecular area occupied by the trabeculae of class 14 (PAR), this parameter decreased significantly in EV-electroporated animals compared to controls (EV-ET compared to CTR: −83%, *p* = 10^−12^, threshold *p* < 0.0021) and further decreased following SOST electroporation, but not significantly (SOST-ET compared to EV-ET: −29%, *p* = 10^−4^, threshold *p* < 0.0014) (Figure 7D). Therefore, micro-CT analyses showed a significant decrease of class 14 trabeculae in mice that underwent electroporation of a plasmid containing the *SOST* gene with respect to both the mice electroporated with an empty vector and the controls, inversely to class 8 trabeculae that showed increased values after treatment.

## 4. Discussion

Since the discovery of myostatin in 1997, the first myokine that has been identified, secretome-based analysis of the human myocyte culture medium has revealed over 600 myokines to date [33,34,35,36,37]. However, the majority of these myokines are still not sufficiently characterized and need to be extensively investigated to gain insight into the pathogenesis of musculoskeletal diseases.

In this study, we proposed to model in vitro the muscle-to-bone communication with osteogenic (2T3) and myogenic (C2C12) cell lines. We found that the conditioned medium from differentiating C2C12 cells significantly altered the functional maturation of osteoblasts. Therefore, we hypothesized the presence of muscle cell-secreted factors that may exert an inhibitory action on osteogenesis. Recently, our group described an osteocyte-produced factor, Wisp2, that is an autocrine/paracrine activator of canonical Wnt signaling pathway with a potent role in accelerating the osteogenesis process [28]. Since sclerostin, an inhibitor of the Wnt/β-catenin pathway that impairs bone formation, has become an attractive therapeutic target for treating osteoporosis, we wondered if this molecule could also be produced by muscle cells. Despite being considered an osteocyte-specific protein, *SOST* mRNA expression was found in other organs, including skeletal muscle [29], but no one had ever characterized its actual synthesis by muscle in detail. Thus, we analyzed SOST/sclerostin protein expression in C2C12 and C57 muscle cells and its secretion into the culture medium, discovering that it is produced at all these levels. We then focused on the characterization of sclerostin in vivo, highlighting that it is widely expressed in young, adult, and old mouse muscles and that its expression undergoes changes in muscles with different metabolic profiles.

In order to determine whether muscular sclerostin was similar to that of osseous sclerostin in the ability to modulate bone remodeling, a transient gain-of-function experiment was performed via plasmid electroporation in muscles of young mice characterized by a growing skeleton. This experiment allowed outlining that muscle-released sclerostin is able to negatively influence the skeletal system. This hypothesis was confirmed by (i) the static histomorphometry performed in the trabecular bone of the femur distal metaphysis (see above); (ii) the micro-CT analysis performed on tibiae, which revealed that SOST overexpression induced a decrease in the number and extension of parallel trabeculae. Diversely, the increased amount of perpendicular trabeculae observed in SOST-ET animals has an explanation related to skeletal homeostasis. As regards the mechanical meaning of trabecular orientation, it is well established that trabecular structures are aligned in an organized manner associated with the direction of load distribution so that trabeculae are mainly oriented according to the principal applied mechanical load directions [38,39]; in line with this evidence, our results on electroporation of the plasmid containing the *SOST* gene show a significant decrease under the effect of muscle-derived sclerostin of bony trabeculae less subjected to increasing load during growth (class 14), differently from the trabeculae (class 8) mostly subjected to increasing load during growth, on which the effect of growing is likely dominant compared to other conditioning factors. Thus, we argue that, particularly in dynamic situations (such as in the growing skeleton) and despite sophisticated analyses (such as micro-CT), the effect of downregulation on osteogenesis exerted by sclerostin of muscular origin can be masked by the effect exerted on the skeleton by the increase in body mass during somatic growth.

Taken together, our data indicate that the action of muscular sclerostin resembles the activity of the osseous one. Therefore, muscular sclerostin seems to behave as an inhibitory factor for osteogenesis that may act synergistically with bone sclerostin to block bone deposition. In suggesting the in vivo role of muscular sclerostin on bone tissue, the authors are aware of the need to confirm the role of muscular sclerostin after the end of the somatic growth before proposing the validation of this protein as a therapeutic target for the treatment of bone processes directly linked to the pathological condition or indirectly, due to a muscular weakness, such as the condition of muscular dystrophy, neuronal atrophy, or cachexia. In line with this target, further steps will be as follows: (i) detailed analyses of the biochemical structure of muscular sclerostin (comparing it with the osseous one) and (ii) investigations on the potential of anti-sclerostin compounds to also inhibit the muscular form.

In conclusion, this study highlights for the first time the presence of a new form of sclerostin produced at the muscular level that can act in combination with bone sclerostin in the phenomena of increased bone fragility. Targeting pathways that centrally regulate bone and muscle or pathways that facilitate communication between the two tissues are the privileged directions for the identification of new chemical molecules, which could prevent, reduce, or restore bone loss.

## Figures and Tables

**Table 1:** The classes of metaphyseal trabeculae were defined by a unique combination of trabecular thickness (pixels) and orientation defined by angle degrees.

Trabecular Class	Thickness (pixels)	Angle (degrees)
1	2	0.0
2	3	0.0
3	2	22.5
4	3	22.5
5	2	45.0
6	3	45.0
7	2	67.5
8	3	67.5
9	2	90.0
10	3	90.0
11	2	112.5
12	3	112.5
13	2	135.0
14	3	135.0
15	2	157.5
16	3	157.5

## Data Availability

Data is contained within the article or supplementary material.

## Supplementary Materials

The following are available online at https://www.mdpi.com/2227-9059/9/1/71/s1, Figure S1: Western Blot C2C12 cells; Figure S2: Western blot C57 cells; Table S1: raw data obtained by experimental procedures showed in Figure 5D–F; Figure S3: Semi- quantification analysis relative to Figure 4B,C.
