# Supplementary material for: Identification of Sclerostin as a Putative New Myokine Involved in the Muscle-to-Bone Crosstalk
Source: Biomedicines. 2021 Jan 12;9(1):71. doi: 10.3390/biomedicines9010071 (PMC7828203; doi:10.3390/biomedicines9010071)
Supplement: Supplementary file 1 [file biomedicines-09-00071-s001.pdf]

SUPPLEMENTARY

Figure S1

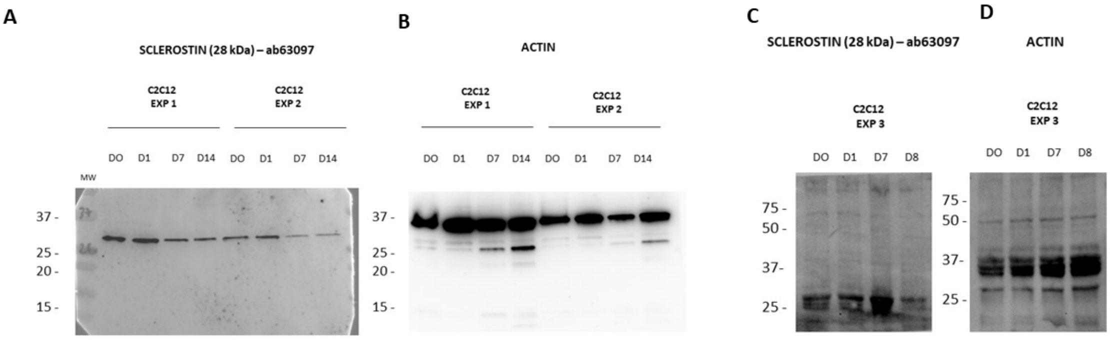

Figure S2

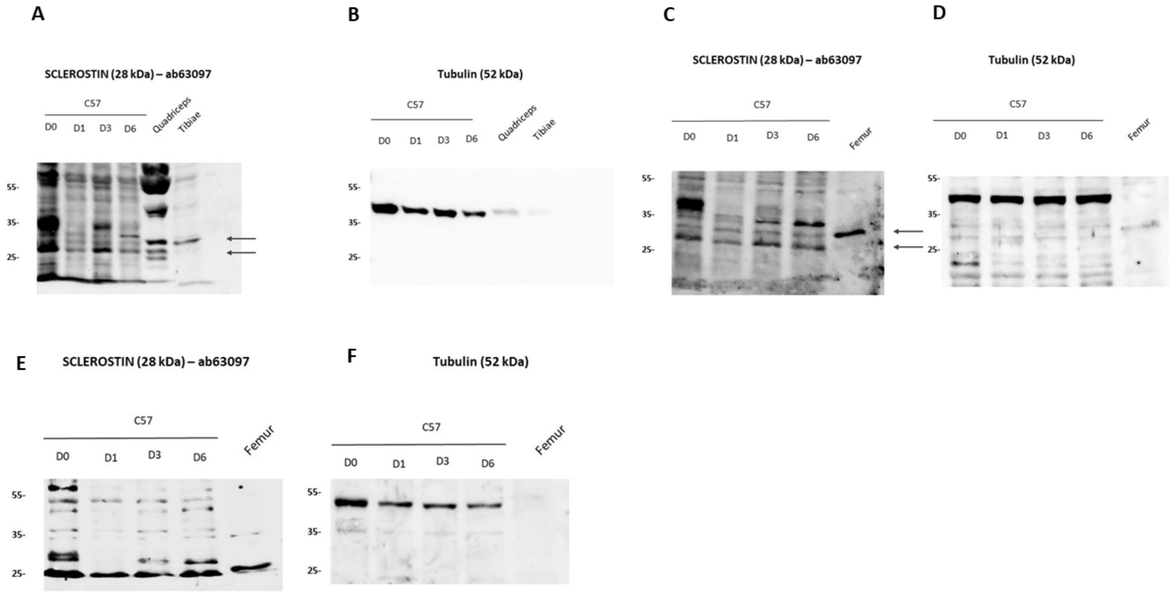

Figure S1-S2: C2C12 and C57 maintained in cycling (DO) and differentiating conditions (D1-D3-D6-D7-D14) were analyzed in western blot. 40ug of protein extracts were resolved in SDS-PAGE and Sclerostin expression was revealed. Where indicated, 40 ug of protein extracts obtained by quadriceps and femur/tibiae were added.

Table S1: Raw data recorded in Fig. 5D-5E-5F were shown in tables.

Sost Myc-Tag								
Tibialis Anterior			Gastrocnemius			Quadriceps		
ET	Empty	NOT ET	ET	Empty	NOT ET	ET	Empty	NOT ET
14,221	0,081	8,168	6,126	0,066	0,940	0,883	0,033	0,815
7,972	0,595	2,694	22,864	0,061	1,670	10,966	0,116	1,986
8,785			7,808	0,434		0,298	0,088	
Average								
10,326	0,338	5,431	12,266	0,187	1,305	4,049	0,079	1,401
SD								
3,398	0,363	3,870	9,216	0,214	0,517	5,998	0,042	0,828

Fig. 5D

Sost								
Tibialis Anterior			Gastrocnemius			Quadriceps		
ET	Empty	NOT ET	ET	Empty	NOT ET	ET	Empty	NOT ET
51,268	0,077	9,815	27,569	0,100	1,098	2,021	0,034	1,068
18,831	0,712	3,458	91,773	0,093	2,282	37,271	0,183	2,395
21,112			42,078	0,586		0,599	0,130	
Average								
30,404	0,394	6,637	53,807	0,260	1,690	13,297	0,116	1,732
SD								
18,105	0,449	4,495	33,670	0,283	0,837	20,775	0,075	0,938

Fig. 5E

Sample ID	Δ [SOST] (pg/ml)	Mean	SD
ET	-32,574	24,794	50,491
	62,478		
	44,478		
Empty	69,120	47,406	19,220
	40,524		
	32,574		
Not ET	-6,354	-22,245	22,473
	-38,136		

Fig. 5F

Figure S3

4B

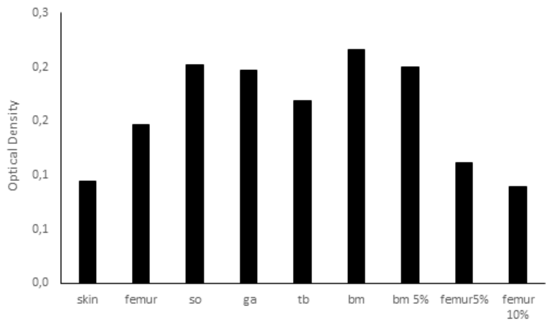

4C

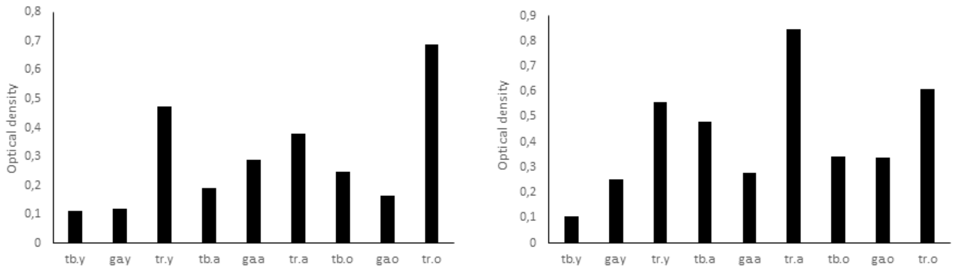

Figure S3: Quantification based on total amount of protein/lane *versus* Sclerostin protein of western blot shown in figure 4B and 4C
